# Active Surveillance for Localized Prostate Cancer – Current Practices and Recommendations

**DOI:** 10.1100/tsw.2010.227

**Published:** 2010-12-14

**Authors:** Jennifer N. Wu, Marc A. Dall'Era

**Affiliations:** Department of Urology, University of California, Davis Medical Center, Sacramento, CA, USA

**Keywords:** prostate cancer, localized, active surveillance, review

## Abstract

Prostate cancer is now the most commonly diagnosed solid tumor in American men, due in part to widespread screening and aggressive diagnostic practices. Prostate cancer autopsy studies show the uniquely high prevalence rates of small, indolent tumors in men dying of other causes. These findings have led to increased concern for the overdetection and overtreatment of prostate cancer. Active surveillance for prostate cancer allows one to limit prostate cancer treatment with concomitant risks of treatment-related morbidity to the men who will benefit the most from aggressive therapies. Several tools have been developed in treated and surveyed men to assist physicians in selecting men with potentially indolent tumors amenable to active surveillance. Recent published results describe institutional experiences with active surveillance and delayed selective therapy for men with low-grade, early prostate cancer. Although median follow-up from these studies is relatively short, the outcomes appear favorable. Data from these reports provide information for selecting men for this approach, as well as for following them over time and determining triggers for further intervention. Ongoing clinical trials with watchful waiting and active surveillance for prostate cancer will ultimately provide improved evidence for managing early, localized disease.

## INTRODUCTION

Prostate cancer is the second leading cause of cancer mortality among men in the U.S., accounting for 29,093 deaths in 2007[1]. It is estimated that there will be 217,730 men diagnosed with prostate cancer in 2010, yet only a fraction of men with localized prostate cancer will ultimately die from their disease[2,3]. Multiple autopsy series describe the substantial rate of clinically undiagnosed and likely indolent prostate cancer, more so than any other solid tumor[4,5]. The incidence of these tumors ranges from 2 to 29% for men in their 30s and 40s, up to nearly 55 to 64% by the 6$^{th}$ and 7$^{th}$ decades of life[6]. The majority of these tumors are well differentiated and these findings are consistent with the prolonged natural history of untreated, low-grade prostate cancer elegantly outlined by Albertsen et al. several years ago[2]. Taken together, these data have fueled a growing concern over the overdetection of prostate cancer, with reported rates as high as 42% in the U.S.[7]. Overdetection refers to the diagnosis of tumors that pose no risk to patient longevity. Overdetection is problematic in that it can lead to overtreatment and cause significant patient anxiety regarding treatment decisions, along with considerable decrements in quality of life. The recent results from the European Randomized Study of Screening for Prostate Cancer (ERSPC) showed a survival advantage with prostate-specific antigen (PSA) screening. However, among the 1,068 men in their screening arm, they estimated that 48 men needed to be treated in order to save one life[8]. Results from the Prostate, Lung, Colorectal, and Ovarian (PLCO) prospective randomized trial on PSA screening showed that prostate cancer death rates did not differ significantly among those in the screening arm and those in the control arm. However, these findings have been criticized due to the high level of prescreening in the control arm[9]. In addition to these two randomized trials, multiple, population-based, observational studies demonstrate the overdetection and overtreatment of low-risk prostate cancer[10,11,12]. Data from these trials prompted the National Comprehensive Care Network (NCCN) and the American Cancer Society to promote active surveillance for certain men with prostate cancer.

The active treatment of prostate cancer by any modality can be associated with decreases in quality of life measures, especially with regard to urinary continence and sexual function[13]. Despite these adverse effects, active treatment remains the mainstay of therapy. In a recent SEER database review, over 75% of patients diagnosed with prostate cancer and PSA <4.0 mg/ml underwent radical prostatectomy or radiation therapy[12]. In an analysis of the CaPSURE database, only 6.8% of patients with localized prostate cancer elected active surveillance[10].

Although the potential quality of life decrements for patients undergoing active treatment for prostate cancer are well documented, men on active surveillance may also be affected. Several studies report that men undergoing watchful waiting may experience considerable anxiety, illness uncertainty, and a decreased quality of life[14,15,16,17]. It remains unclear, however, whether these effects are better or worse than those experienced by men who have been treated radically. It appears that most patients with prostate cancer, whether treated or not, are concerned about the risk of progression and anxiety about PSA recurrence. It is important to be aware of the psychosocial impact that patients on active surveillance may experience and council them accordingly. Avoidance and control of disease anxiety has also been shown to impact surveillance adherence and selection of radical therapy.

The central tenet behind active surveillance for prostate cancer is that some men with prostate cancer may delay or avoid treatment for tumors that pose no risk to their longevity. This approach relies on careful risk stratification schemes that are continually being improved in order to identify patients with a low risk of cancer progression, using clinical variables to further stratify patients in the low-risk group into even lower-risk groups[18].

## DEFINING LOW-RISK PROSTATE CANCER

Due to the heterogeneous nature of prostate cancer, it is important to select the appropriate group of patients that benefit the most from deferring active treatment without compromising outcomes. The categorization of patients into this low-risk group is challenging and likely one of the primary obstacles to more widespread acceptance of active surveillance. Multiple tables and nomograms integrating clinical variables, such as Gleason score, pretreatment PSA, clinical stage, and pathological data, assist patients and physicians to estimate the risk of long-term prostate cancer progression and disease-specific mortality primarily for treated men. One of the most popular and well-recognized nomograms in prostate cancer by Kattan et al. can be used to predict prostate tumors that are likely to be indolent in nature based on pathological criteria at the time of prostatectomy[19]. For the Kattan nomogram, indolent disease is defined as organ-confined, well-differentiated prostate cancer measuring under 0.5 ml in volume, estimated by step sectioning the specimen after radical prostatectomy. For nomogram generation, the authors found that 23% of tumors in the dataset were indolent. This nomogram has since undergone external validation and recalibration for a PSA-screened population utilizing the ERSPC dataset. In this analysis, 485 men were predicted to have indolent cancer, representing 30% of screen-detected men[20].

The validated Cancer of the Prostate Risk Assessment (CAPRA) score is a useful tool to help further substratify patients in the low-risk classification in terms of likelihood of disease progression and has been shown to predict cancer-specific mortality primarily in treated men[18,21]. These tools, however, provide only population-based predictions and do not offer individual patient-based risk assessment for personal decision making.

The Epstein criteria, first described in 1994 and then updated in 2004, integrate pathologic criteria along with clinical data to identify potentially low-risk tumors and are a commonly used method to describe disease risk[22,23]. The criteria were developed for men treated with radical prostatectomy with a strict pathologic description for what was considered indolent disease: tumor size <0.5 $cm^3$, no disease outside the prostate, and no Gleason pattern 4 or 5. The preoperative predictors associated with these "insignificant" tumors include no Gleason pattern 4 or 5 in the biopsy specimen and either (1) PSA density of 0.1 ng/ml/g or less, fewer than three positive biopsy cores (minimum of six cores taken), and no cores with >50% involvement; or (2) PSA density of 0.15 ng/ml/g or less and cancer smaller then 3 mm on only one biopsy core (minimum of six cores taken). Although the Epstein criteria have not necessarily been shown to predict more robust outcomes such as disease-specific mortality, it is widely accepted and used today to identify low-risk tumors.

In 1998, D'Amico et al.[24] described another risk classification scheme for prostate cancer using clinical stage, pretreatment PSA, and Gleason score to place patients in low, intermediate, or high risk of PSA recurrence after primary radiotherapy or radical prostatectomy. This classification was then updated to include estimates of tumor volume from the diagnostic biopsy[25]. The D'Amico criteria have been validated with other datasets and were shown to predict disease-specific and all-cause mortality in men undergoing radical prostatectomy[26]. It is important to recognize that both the Epstein and D'Amico criteria were developed and designed to predict outcomes in the prostate cancer of treated men. Both means of classification are widely accepted, however, and partly due to ease of use, most commonly employed in selecting men with likely indolent disease for active surveillance.

In 2007, Kattan et al. developed the first nomogram using data from *untreated* men with prostate cancer to predict the more robust outcomes of disease-specific and overall mortality[27]. This dataset was obtained from six British cancer registries and included over 1,100 men with 431 prostate cancer–specific deaths. In this analysis, biopsy Gleason grade was the greatest predictor of disease-specific survival and the nomogram performed well with concordance indices of 0.73 to 0.74. In this analysis, 24% of men died from prostate cancer within 10 years of diagnosis. However, the men were not under a defined surveillance protocol and were not uniformly monitored for early progression.

In addition to using nomograms to define low-risk prostate cancer, there are emerging techniques in magnetic resonance imaging with spectroscopic imaging (MRI/MRSI) that may improve prostate cancer detection and characterization[28]. This technology integrates anatomic with molecular data and assesses tumor metabolism, which may aid in the prediction of tumor aggressiveness or progression for active surveillance protocols. Shukla-Dave et al. developed a nomogram for predicting clinically insignificant prostate cancers by incorporating MRI/MRSI findings with clinical variables[29]. Prando et al. transferred data from MRI to transrectal ultrasound imaging to sample regions of cancer in men with rising PSA levels and negative findings at prior biopsy with good accuracy[30]. A recent retrospective study by Fradet et al. from UCSF determined that patients on active surveillance with MRI/MRS findings suggestive of cancer at initial diagnosis had a greater risk of Gleason score upgrade at subsequent biopsy[31]. These studies suggest a potential role for prostate MR in identifying and counseling potential patients about active surveillance. These new technologies must be tested rigorously in larger, prospective studies before being relied upon for predicting clinically meaningful tumors or candidacy for active surveillance.

## SELECTING PATIENTS FOR ACTIVE SURVEILLANCE

Active surveillance protocols attempt to identify men with a good risk for prostate cancer who are most likely to be safely watched for a period of time and then treated when necessary. Inclusion criteria into active surveillance programs vary by institution and are based largely on personal preferences and individual clinical experiences with no hard data.

Multiple clinical variables should be integrated to estimate risk, and to guide timing and choice of treatment. Pathological biopsy results play a large role in decision making and should be based on a well-performed, 12-core-minimum biopsy with anterior gland sampling. Many centers offer active surveillance primarily to men with clinically localized disease (cT1–T2), Gleason score <6 with no pattern 4 or 5, and PSA <10. In addition, biopsy estimates of disease volume are also considered, including the number or fraction of total biopsy cores positive and the percentage of individual cores occupied with cancer. Both the Epstein and D'Amico criteria for defining disease risk are commonly used for selecting candidates for surveillance and have been used for selecting men for entry into clinical trials.

Several centers have advocated use of a "confirmatory" prostate biopsy before selecting active surveillance for treatment, as contemporary methods of transrectal prostate biopsy have a well-recognized risk of understaging and undergrading prostate cancer. Our ability to assess tumor grade and volume accurately is critical when making prostate cancer treatment decisions. A radical prostatectomy series of men considered candidates for active surveillance showed a 28–46% risk of clinical undergrading with prostate biopsy, which draws obvious concern for men making treatment decisions based on initial biopsy results[32,33]. In concordance with these findings, data from Memorial Sloan Kettering show that immediate prostate rebiopsy in patients considered candidates for active surveillance resulted in 27% having higher grade or volume disease[34]. Additionally, these patients were more likely to show higher grade and stage disease at radical prostatectomy. Early repeat prostate biopsy may, therefore, serve to limit undersampling and improve discrimination of who the best candidates are for active surveillance with selective delayed intervention. In general, it is felt that grade progression within a few years of diagnosis more likely represents clinical undersampling over actual dedifferentiation of the tumor, so this early "confirmatory" biopsy can detect higher-grade cancers earlier for more appropriate therapy[35].

There is considerable interest and research aimed at improving techniques for prostate biopsy, as there are clear imitations to standard transrectal ultrasound–guided approaches. Prostate saturation biopsy utilizing a perineal approach is being investigated to enhance the detection and characterization of prostate tumors. A review of the literature on biopsy strategies by Patel and Jones advocates the use of saturation biopsy for the restaging of patients undergoing active surveillance protocols, but not for the initial biopsy[36]. Abouassaly et al. investigated the use of saturation biopsy for restaging men in their active surveillance protocol[37]. At a median of 9 months, 38% of patients were upstaged as defined by an increase in Gleason score or tumor volume. Patients who were initially diagnosed with prostate cancer using extended systematic biopsy had a five times higher risk of upstaging and/or upgrading than those who were diagnosed with prostate cancer via saturation biopsy (11% with 20 cores or more compared with 55% with fewer than 20 cores). In contrast, Fleshner and Klotz did not find value in using saturation techniques for repeat prostate biopsy, although their patient population underwent saturation biopsy after having more than three negative prostate biopsies[38]. Other technologies, such as three-dimensional mapping biopsies, may not only improve cancer grade and stage prediction, but may also provide the ability to precisely rebiopsy tumors within the prostate to track them over time[39].

The 5-alpha reductase inhibitors finasteride and dutasteride may have a role for improving the diagnostic yield of prostate biopsy for patients on active surveillance. Results from the Prostate Cancer Prevention Trial (PCPT) showed that finasteride taken orally at 5 mg/day reduced the overall risk of being diagnosed with prostate cancer by 25% compared with placebo. The study also showed, however, a greater incidence of high-grade tumors in the men taking finasteride[40]. Subsequent follow-up analysis of this study offered a possible explanation for these findings in that the finasteride-treated patients had significantly smaller prostates, resulting in a disproportionate sampling of the tumor during biopsy, thus increasing the sensitivity of the biopsy to detect high-grade tumors[41]. Men with large prostates may therefore benefit by gland volume reduction to increase the sensitivity of surveillance biopsies. However, the utility of this remains unknown. In addition, the effect of 5-alpha reductase inhibitors on serum PSA can skew PSA kinetic calculations. The Reduction by Dutasteride of Clinical Progression Events in Expectant Management (REDEEM) study is a phase III trial testing the effect of dutasteride on the secondary prevention of prostate cancer progression for men on active surveillance[42]. When available, data from this trial will better define the role of these medications in the management of prostate cancer.

The criteria for inclusion into active surveillance vary by institution, but typically include some combination of the factors listed in Table 1. Although not commonly reported on, PSA velocity (PSAV) before treatment has been shown to be predictive of outcome after radical prostatectomy or external beam radiation, and may be useful in evaluating patient risk as well[43]. In addition to the factors mentioned, age (<55 or >75 years), family history, and medical comorbidities should be taken into account when counseling and selecting patients for active surveillance.

**TABLE 1**
**Active Surveillance Inclusion Criteria**

- PSA <10 or <15 ng/ml
- Clinical stage T1c or ≤T2a or ≤T2b
- Gleason score ≤6 or ≤7 (3+4)
- Positive cores <3 or <33% of total cores
- Maximum percent of cores having cancer <50%
- PSAD <0.15 or <2.0 ng/ml
- Patient psychologically committed

## ACTIVE SURVEILLANCE OUTCOMES: SURVEILLANCE STRATEGIES AND TRIGGERS FOR INTERVENTION

Several institutions have published results on their active surveillance series with promising findings (Table 2). Parulekar et al.[44] reported long-term follow-up of their prospective series of 450 patients. Entry criteria in this series include men with clinically localized disease with Gleason grade 3+3 and some with low-volume 3+4. Epstein's criteria for predicting insignificant disease were used for men younger than age 55. Men were followed with PSA measurement every 3 months for the first 2 years and then every 6 months. Surveillance biopsies were done initially 6–12 months after diagnosis and then every 3–4 years. Clinical progression in this series is primarily defined biochemically, with a PSA doubling time of <3 years used as the principal trigger for intervention (Table 3). Men with Gleason grade changes to 4+3 or greater on surveillance biopsy or other clear evidence of disease progression were also recommended for treatment. The 10-year prostate cancer actuarial survival was 97.2% in this series and, with a mean follow-up of 6.8 years, 30% of the men have progressed. Of the 117 patients eventually treated with radical prostatectomy, 50.4% had PSA failure. This clearly represents a high and concerning biochemical failure rate; however, it only accounts for 13% of the entire cohort. On multivariate analysis, PSA doubling time of 3 years or less was associated with an 8.5 times increased risk of PSA failure after definitive therapy in this group of men. The hazard ratio for non–prostate cancer to prostate cancer death mortality was 18.6 at 10 years. In a follow-up paper describing the characteristics of the five men who died of prostate cancer after a period of active surveillance, the authors found that three of the five men had favorable-risk disease at entry, but all had rapid PSA doubling time of <1.6 years[45]. It is important to bear in mind that men undergoing surgery after active surveillance in these series likely represent a select higher-risk group at baseline. It is difficult to compare these to a cohort of similarly risk-stratified men at baseline undergoing immediate surgery as these surgical series are "diluted" by many truly low-risk men.

Investigators from the University of California, San Francisco[46] reported a series of 321 men on active surveillance as initial management for prostate cancer. In general, entry criteria for surveillance included men with Gleason 3+3, cT1–T2 disease, PSA <10, ≤1/3 biopsy cores positive, and ≤50% single core involvement. With a median follow-up of 3 years, 24% of men received secondary treatment with changes in Gleason score on subsequent biopsy being the greatest driver for recommending treatment. Also noted was that 13% of patients in this series came off of surveillance and selected active treatment

**TABLE 2**
**Active Surveillance Cohorts**

| Study | N | Median Follow-Up (Years) | Eligibility Criteria | Treatment Criteria | Treated |
|---|---|---|---|---|---|
| Toronto[44] | 450 | 6.8 | Gleason score ≤6; PSA ≤10 ng/ml; **for patients ≥70 years old:** Gleason score ≤3+4 PSA ≤15 ng/ml | PSA DT <3 years; Gleason grade ≥7; cT3 | 30% |
| Sweden[52]) | 6,849 (2,686 low-risk group) | 8.2 | Gleason score ≤7; PSA <20 ng/ml; patients <70 years old; clinical T1-2 | Variable | 34% |
| Hopkins[48] | 407 | 3.4 | PSAD <0.15 ng/ml/cm$^3$; Gleason score <6 (no pattern 4 and 5); <2 cores involved; <50% of any 1 core involved; clinical T1c | Gleason score >7 on rebiopsy (any pattern 4 or 5); >2 cores involved; >50% of any 1 core involved | 25% |
| MSK/Baylor[36] | 88 | 3.7 | Gleason score increase; PSAV >0.75/year; increase DRE/TRUS-detected lesion; increase biopsy volume | Score based on: Gleason score increase; PSAV >0.75 ng/ml/year; increase DRE/TRUS-detected lesion; Increase biopsy lesion | 35% |
| Miami[56] | 99 | 3.8 | Gleason sum ≤6; PSA ≤15 ng/ml; clinical stage ≤T2; ≤50% of two biopsy cores | Rising PSA, PSA DT; Gleason sum ≥7 on rebiopsy; increase in tumor volume; stage progression; patient preference | 8% |
| UCSF[46] | 321 | 3.6 | PSA <10 ng/ml; Gleason sum <6 (no pattern 4 or 5); <33% cores involved; clinical T1/T2a | Rising PSA (PSAV >0.75 ng/ml/year); Gleason sum ≥7 on rebiopsy; increase in volume by biopsy parameters | 24% |

**TABLE 3**
**Active Surveillance Trigger for Intervention**

- Increase in Gleason score ≥7 (3+4 or 4+3)
- PSAV >0.75 or >1 ng/ml/year
- PSA doubling time <3 years
- Cores involved >2
- Percent of cores having cancer >50%
- Clinical judgment
- Change in DRE
- Patient preference without evidence for disease progression. This shows that a not insignificant number of men become unwilling or unable to tolerate active surveillance. At an overall median follow-up of 3.6 years, the disease-specific survival rate was 100%. In a group of men undergoing radical prostatectomy, a median of 18 months after diagnosis, final pathologic outcomes including rates of extracapsular extension and seminal vesicle involvement did not appear compromised by the initial period of surveillance[47].

Data on a cohort of over 400 men from Johns Hopkins University[48] show that after mean follow-up of 2.2 years, 25% of men underwent curative intervention. These men all met Epstein's criteria upon initiation of active surveillance, and were followed with PSA measurement and rectal exam every 6 months with repeat prostate biopsies every year. Older age at diagnosis was significantly associated with active treatment. When analyzing men with at least one follow-up biopsy after diagnosis, 32.7% had progression through an unfavorable biopsy at a median follow-up of 5.6 years[49]. Progression was primarily defined as Gleason pattern 4 or 5, greater than two biopsy cores with cancer, or >50% involvement of any core with cancer. The authors found on multivariate analysis that initial biopsy core involvement ≥35% and %free PSA ≤15% were predictive of progression. A second analysis was done to assess the utility of PSA kinetics in predicting adverse pathologic features on surveillance biopsy[50]. While PSA doubling time showed no correlation, there was a statistically nonsignificant median PSAV difference of 0.2 and 0.5 ng/ml/year between those who did not and did progress, respectively. The authors were not able to define a clinically useful PSAV cutoff that could predict disease progression on surveillance biopsy. Other studies have shown a significant relationship between PSAV and adverse histology on subsequent biopsy[51].

The National Prostate Cancer Registry of Sweden Follow-Up Study reported results on 2,686 men with low-risk prostate cancer (clinical T1c, Gleason score 2–6, and serum PSA <10 ng/ml) who were managed expectantly with either active surveillance or watchful waiting[52]. After a median follow-up of 4 years, 34% of the men received treatment. The 10-year prostate cancer–specific mortality was 2.4% among patients with low-risk prostate cancer in the active surveillance group compared to 0.4% in the radical prostatectomy group and 1.8% in the radiation therapy group. Death due to competing causes was 19.2% for the active surveillance group, 8.5% for the radical prostatectomy group, and 14.2% for the radiation therapy group, demonstrating how, in general, men treated with expectant management had more comorbidities. Although this study had less stringent inclusion criteria than other published series, it suggests that active surveillance is a suitable treatment option for patients with low-risk prostate cancer.

In summary, these series show that in the short term, active surveillance for prostate cancer is feasible with 20–30% of men showing evidence of progression within 2–3 years. The role of PSA kinetics in monitoring men with disease remains unclear. Most centers will advocate early confirmatory biopsy for entry into active surveillance with up to 30% of men having higher grade or volume disease found. Up to 50% of men may not have any cancer found on early rebiopsy.

## THE FUTURE DIRECTIONS OF ACTIVE SURVEILLANCE

Although these mostly single-institution experiences are very promising, results from current ongoing large randomized trials comparing expectant management or watchful waiting to active treatment, such as PIVOT[53], START[44], and ProtecT[54], will validate and confirm many of the criteria for patient selection and monitoring. The Prostate Cancer Intervention versus Observation Trial (PIVOT) specifically enrolled an ethnically diverse cohort and will compare outcomes between radical prostatectomy and traditional watchful waiting[53]. The Study of Active Surveillance Therapy against Radical Treatment in Patients Diagnosed with Favorable Risk Prostate Cancer (START) will offer patients, in the randomly assigned active treatment group, a choice between radical prostatectomy or radiation[44]. The Prostate Testing for Cancer and Treatment (ProtecT) feasibility study aims to enroll 2,000 men and will compare external beam radiation, prostatectomy, and active monitoring[54]. Men with rapid PSA doubling times will be allowed active therapy in this trial. The CANARY trial is a multicenter study and biorepository attempting to identify and confirm biomarkers of aggressive disease as defined by prespecified histologic, PSA, and clinical criteria[55]. The participants in this study have already chosen active surveillance as their preferred treatment plan before enrollment. A central aim of this trial is to identify biomarkers that could be used for patient selection as well as disease progression in order to target treatment for select men and provide better insight into the natural history of prostate cancer.

Until data from these active surveillance trials are available, treatment decisions regarding active surveillance for localized prostate cancer will vary between physicians. In general, inclusion criteria should include a low (<10 ng/ml) and stable PSA, Gleason grade ≤6, clinical stage T1–T2a, and low-volume disease as assessed by extended-pattern (12 or more cores) biopsy. Men should be followed closely with PSA measurements every 3–4 months, digital rectal exams every 3–6 months, and imaging (if performed) every 9–12 months. Repeat "confirmatory" prostate needle biopsy should be considered within a few months of diagnosis, after 1 year of surveillance, and then every 12–24 months, or as indicated by changes in PSA or findings on exam. Active surveillance offers men the opportunity to delay active treatment and its associated morbidities until evidence of clinical progression is found.
